# Quantitative assessments of late radiation-induced skin and soft tissue toxicity and correlation with RTOG scales and biological equivalent dose in breast cancer

**DOI:** 10.1007/s12094-021-02729-z

**Published:** 2021-11-18

**Authors:** Y. Huang, J. Sanz, N. Rodríguez, X. Duran, A. Martínez, X. Li, P. Foro, M. Conde, M. Zhao, F. Liu, A. Reig, J. Dengra, I. Membrive, P. Pérez, M. Algara

**Affiliations:** 1 Autonomous University of Barcelona, Barcelona, Spain; 2 Radiation Oncology Department, Hospital del Mar, Parc de Salut Mar, Barcelona, Spain; 3 Radiation Oncology Research Group, Institut Hospital del Mar d’Investigacions Mèdiques, Barcelona, Spain; 4 Statistics Department, Institut Hospital del Mar d’Investigacions Mèdiques, Barcelona, Spain; 5 Radiation Oncology Department, Hospital del Mar, Edifici B, c/Gas s/n Planta-2, 08003 Barcelona, Spain

**Keywords:** Radiation-induced toxicity, Biological equivalent dose, Quantitative assessment, Objective evaluation, Breast cancer, Radiotherapy

## Abstract

**Purpose:**

Radiation-induced toxicity (RIT) is usually assessed by inspection and palpation. Due to their subjective and unquantitative nature, objective methods are required. This study aimed to determine whether a quantitative tool is able to assess RIT and establish an underlying BED-response relationship in breast cancer.

**Methods:**

Patients following seven different breast radiation protocols were recruited to this study for RIT assessment with qualitative and quantitative examination. The biologically equivalent dose (BED) was used to directly compare different radiation regimens. RIT was subjectively evaluated by physicians using the Radiation Therapy Oncology Group (RTOG) late toxicity scores. Simultaneously an objective multiprobe device was also used to quantitatively assess late RIT in terms of erythema, hyperpigmentation, elasticity and skin hydration.

**Results:**

In 194 patients, in terms of the objective measurements, treated breasts showed higher erythema and hyperpigmentation and lower elasticity and hydration than untreated breasts (*p* < 0.001, *p* < 0.001, *p* < 0.001, *p* = 0.019, respectively). As the BED increased, Δerythema and Δpigmentation gradually increased as well (*p* = 0.006 and *p* = 0.002, respectively). Regarding the clinical assessment, the increase in BED resulted in a higher RTOG toxicity grade (*p* < 0.001). Quantitative assessments were consistent with RTOG scores. As the RTOG toxicity grade increased, the erythema and pigmentation values increased, and the elasticity index decreased (*p* < 0.001, *p* = 0.016, *p* = 0.005, respectively).

**Conclusions:**

The multiprobe device can be a sensitive and simple tool for research purpose and quantitatively assessing RIT in patients undergoing radiotherapy for breast cancer. Physician-assessed toxicity scores and objective measurements revealed that the BED was positively associated with the severity of RIT.

**Supplementary Information:**

The online version contains supplementary material available at 10.1007/s12094-021-02729-z.

## Introduction

The incidence of breast cancer has increased in recent years and is expected to continue to rise in the next decade [1]. Radiation therapy (RT) is an important complementary treatment that improves local–regional control and reduces the risk of cancer recurrence [2]. However, despite the advances in radiotherapy planning and treatment technology, approximately 30–40% of irradiated patients will suffer late RIT, with complications including dermatitis, fibrosis, desquamation (moist or dry) and even necrosis. As a result, RIT may affect the function of the skin and appearance of the breast, key factors impacting patient satisfaction and quality of life. Nevertheless, as exemplified by the high 5- and 10-year survival rates observed over the past decade (approximately 90% and 80%, respectively), patients could live for many years with RIT [3].

With improvements in radiotherapy planning, many different fractionation schedules and techniques have been applied in clinical practice over the past decade, ranging from classical doses of 2 Gy to new standard daily moderate hypofractionations of 2.7–2.85 Gy [4], or more foreshortened treatment of 26 Gy in 5 consecutive fractions [5]. Accelerated partial-breast irradiation (APBI) is also accepted as an attractive treatment strategy and has been introduced into clinical practice, shortening the duration of treatment and the extent of the irradiated volume, however, the toxicity outcomes are not yet clear and variable between different studies [6–8].

In most previous studies and in current clinical practice, RITs are classified with common rating criteria, such as the Common Terminology Criteria for Adverse Events (CTCAE) and Radiation Therapy Oncology Group (RTOG) scales. These assessments are subjectively carried out by physicians with visual inspections and palpation examinations. Although fast and simple, such qualitative assessments are limited to 4 or 5 discrete grades [9]. In addition, due to its inherently subjective nature, the estimation of skin changes by different physicians is inevitably subjected to interobserver and intraobserver variability and may lead to a nonnegligible bias, particularly in multicenter studies [10].

To avoid this bias, many objective assessment tools have been introduced for monitoring skin changes more accurately, including ultrasound [11–13], reflectance spectrophotometry [14–16], thermal images [17], laser Doppler flowmetry (LDF) [18] and other multiprobe devices which consists of various probes that can assess different skin parameters, including erythema, pigmentation, hydration; skin pH, skin temperature etc. [19–21]. Although these objective techniques demonstrate advantages in providing a more reliable quantification of RIT over subjective assessments, very rare tools have been routinely used in clinical practice or in comparison to RTOG or CTCAE scales successfully.

Furthermore, the dose, fractionation and dose per fraction are considered to impact the RT results. To directly compare the RITs resulting from different radiotherapy regimens, the biological equivalent dose (BED) can be used to more properly directly compare the RIT than a simple dose–response [22].

This study aimed to (1) determine whether our quantitative and multiprobe technique is capable of assessing late RIT in terms of skin color alterations (erythema, hyperpigmentation), induration and dehydration following different radiotherapy protocols; (2) establish an underlying BED-response relationship based on both objective measurements and subjective RIT evaluations; (3) determine whether the measures of our objective assessment tool is related to a subjective clinical assessment of late RIT obtained using the RTOG scale.

## Materials and methods

### Patients

Patients were recruited to this study for RIT assessment with qualitative and quantitative examinations. Patients were recruited if accomplished the following inclusion criteria: age > 18 years old, treatment with unilateral breast radiotherapy, follow-up > 12 months, no antecedent irradiation to the breast or thorax, and acceptance of subjective and objective toxicity assessment. The exclusion criteria were: short follow-up (< 12 months), treatment with bilateral radiotherapy, prior breast or thoracic radiotherapy, pre-existing skin diseases, skin alterations caused by another treatment, refusal of allocated treatment, absence of toxicity assessments, and withdrawal of consent. Study was approved by Ethic Committee. All patients provided a written informed consent for inclusion in the study.

### Radiotherapy and BED

Patient descriptions and fractionation schedules are presented in Table 1. Patients were treated by different sequential radiation protocols with comprehensive nodal plan according to the guideline or research trials performed in our department. To compare the RITs resulting from different fractionation regimens, the linear-quadratic (LQ) model was adopted to calculate the BED for each radiation schedule. An *α*/*β* ratio of 3 Gy for late toxicity of breast tissue was used to calculate the BED from different radiation schemes. Patients were grouped according to the radiation BED used, and a total of 7 groups (A–G) of patients were recruited into our study. Given the BEDs calculated above, different radiotherapy treatment schedules could be directly compared.

**Table 1:** Patient characteristics

Group	Patients. no. (*n*, %)	Radiation regimens	WBI o PBI	BED^#^	Age Mean years (± SD)	Interval time^*^ Mean years (± SD)	Chemotherapy (*n*, %)	Hormonotherapy (*n*, %)
A	25 (12.6)	30 Gy/5 Fx	WBI	78.4	81.8 (± 6.6)	3.4 (± 1.4)	6 (24.0)	21 (84.0)
B	28 (14.1)	48 Gy/2 Fx	WBI	79	58.2 (± 10.9)	3.3 (± 1.9)	17 (60.7)	19 (67.9)
C	40 (20.1)	37.5 Gy/3.75 Fx (BID)	PBI	82.9	66.6 (± 6.0)	3.2 (± 1.9)	2 (5.0)	40 (100)
D	23 (11.6	48 Gy/2 Fx + 10 Gy boost	WBI	95.4	66.1 (± 8.8)	3.0 (± 1.9)	3 (13.0)	21 (91.3)
E	50 (25.1)	40.05 Gy/2.67 Fx + 16.02 Gy boost	WBI	104.2	63.7 (± 7.9)	3.3 (± 0.6)	24 (48.0)	43 (86.0)
F	11 (5.5)	48 Gy/2 Fx + 20 Gy boost	WBI	111.9	54.9 (± 12.6)	3.3 (± 2.1)	5 (45.5)	8 (72.7)
G	17 (8.5)	37.5 Gy/6.25 Fx	WBI	113.1	83.7 (± 6.6)	9.1 (± 2.9)	5 (29.4)	16 (94.1)

*BED* biologically equivalent doses, *WBI* whole-breast irradiation, *PBI* partial-breast irradiation

^#^BED of late toxicity (*α*/*β* = 3.1 Gy), *Interval time (years) between radiotherapy and toxicity assessment

### Clinical toxicity assessment

Physicians subjectively evaluated the patients for late RIT using the RTOG scoring system. Late toxicity was assessed at least 12 months after finishing RT. The examinations were carried out by visual inspection and palpation of both breasts, and the results ranged from grade 0 (no reaction) to grade 4 (severe toxicity).

### Objective quantitative toxicity assessment

**Fig. 1:**
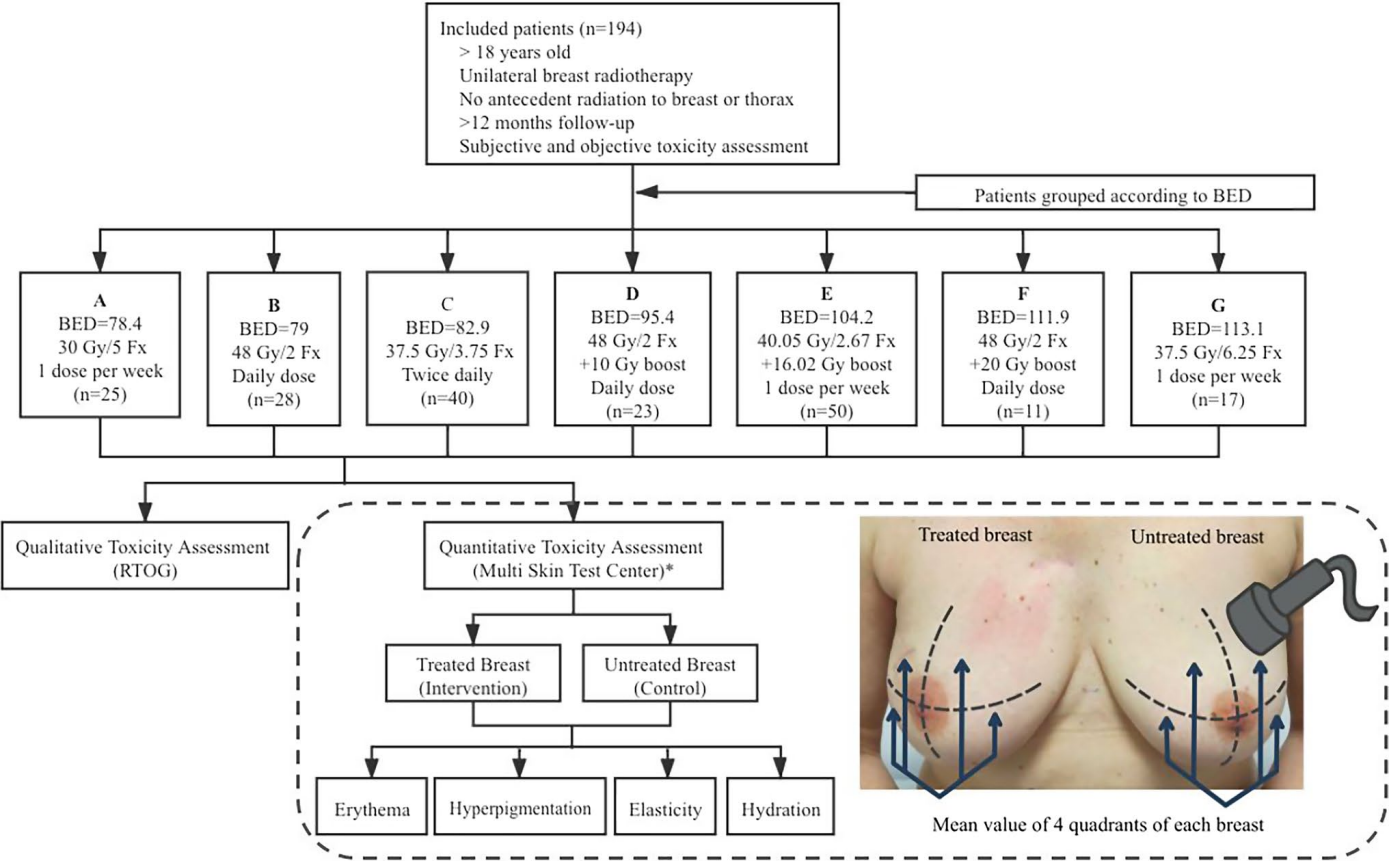
Study design and flow chart of participating patients. *BED* biological equivalent dose; *RTOG* Radiation Therapy Oncology Group. *Quantitative assessment of toxicity by Multi Skin Test Center: measurements were obtained from 4 quadrants of each breast, separately in the irradiated breast and the corresponding nonirradiated breast

A Multi Skin Test Center MC 750 B2 device (CK Electronic, GmbH; Cologne, Germany) was used to simultaneously detect RIT. This multifunctional device consists of various probes that assess four skin parameters for each patient: a mexameter probe for assessing erythema (redness) and hyperpigmentation, a suction cup probe for assessing elasticity (as the surrogate of fibrosis) and a corneometry probe for assessing skin hydration (the relative quantity amount of water on the breast skin). For whole-breast irradiation, measurements were obtained from 4 quadrants of each breast; for partial-breast irradiation, measurements were obtained from 4 separate points of irradiated breast areas (high-dose areas). The measurements separately in the irradiated breast and the corresponding symmetric regions in the nonirradiated breast (Fig. 1). The average toxicity values of all these 4 points of each breast with computerized processing reflect the overall characteristics of the skin and subcutaneous tissues. The entire process of assessing toxicity took approximately 5 min per patient. To exclude the bias of the individual skin quality on the toxicity result, we used the absolute difference in toxicity between the treated and untreated breasts to assess the outcomes (Δerythema, Δpigmentation, Δelasticity and Δhydration).

The results of the objective assessments were also used to determine whether they are correlated with those of the subjective RTOG evaluations. Among them, skin RTOG toxicity scale was used to determine whether erythema and hyperpigmentation were related to subjective clinical assessment; subcutaneous tissue RTOG criteria was used to determine whether elasticity was correlated to physician-assessed toxicity grade.

### Other RIT-related factors

To detect the other potential RIT-related factors, age, interval time between radiotherapy and toxicity measurement, chemotherapy and hormonal therapy were included in regression models. The variables were selected due to clinical relevance. Due to collinearity, those RT-related factors, such as boost, did not include in multivariate analysis.

### Statistics

The BED and toxicity values are presented as the mean with standard deviation and medians with interquartile ranges. A Wilcoxon signed rank test was used to evaluate the significance of the difference between radiated and nonirradiated breasts. A Spearman correlation coefficient and its significance test were used to identify the relationship between the subjective and objective assessment and determine whether RIT is associated with BED. The adjusted associations of radiation schemes and RTOG toxicity scores were studied by ordered logistic regression analysis. Multivariate median regression analysis was used to identify potential predictors of objectively evaluated toxicity: erythema, hyperpigmentation, elasticity and hydration. The statistical analysis was performed with Stata (version 15.1; College Station, TX: StataCorp LLC). *p* < 0.05 was considered statistically significant.

## Results

A total of 194 patients were recruited in this study. Radiation-related information and patient recruitment flow chart are shown in Fig. 1.

**Fig. 2:**
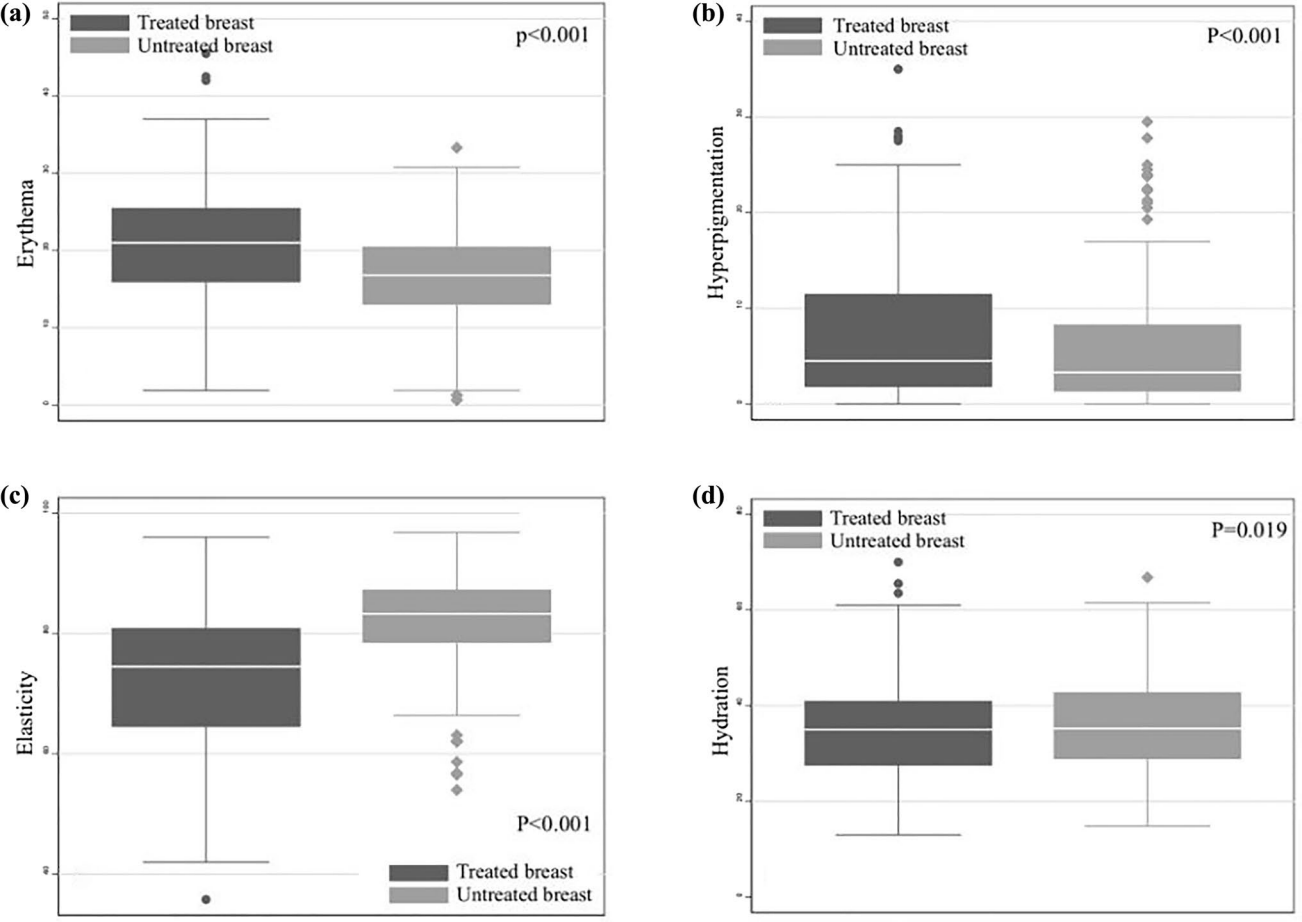
Comparison of erythema (**a**), hyperpigmentation (**b**), elasticity (**c**) and hydration values (**d**) between irradiated and nonirradiated breasts

**Fig. 3:**
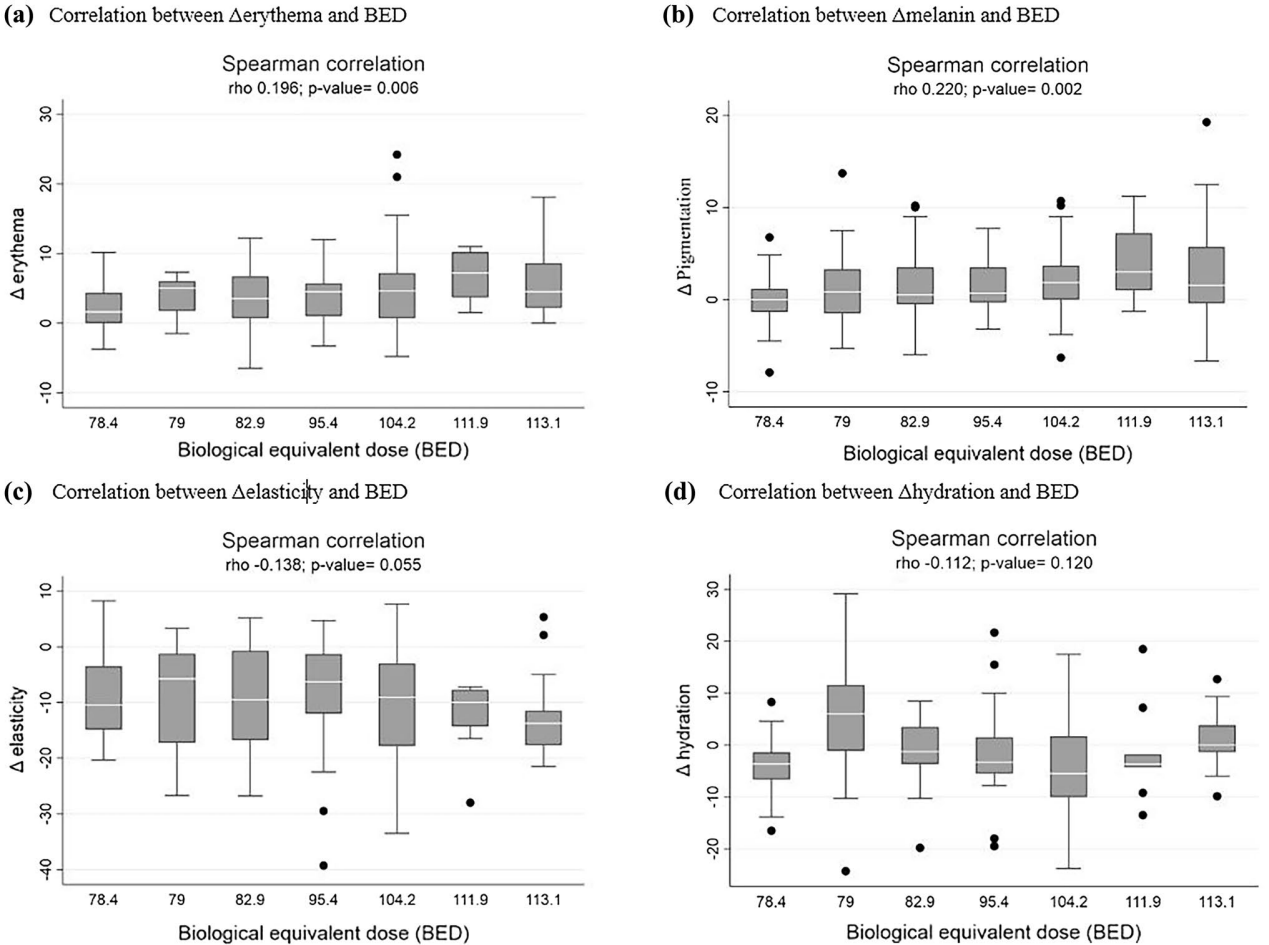
Biological equivalent dose (BED) dependence of Δerythema (**a**), Δpigmentation (**b**), Δelasticity (**c**) and Δhydration (**d**) in patients treated with different radiotherapy protocols. Δ = change of toxicity value between treated and untreated breast

In the comparison of RITs between irradiated and nonirradiated breasts by multiprobe quantitative evaluation, as shown in Fig. 2, the treated breast showed significantly higher redness and hyperpigmentation values than the untreated breast: [median 21.0 (range 15.9–25.6) vs. 16.8 (range 12.9–20.5), *p* < 0.001; 4.5 (range 1.7–11.5) vs. 3.3 (range 1.3–8.4), *p* < 0.001, respectively]. The irradiated breast had a greater loss of elasticity than the nonirradiated breast: median 74.5 (range 64.5–80.9) vs. 83.3 (range 78.4–87.3), *p* < 0. 001. There was a similar but significantly different hydration index between the treated breast and untreated breast [median 35.0 (range 27.5–41.1 vs. 35.2 (range 28.8–42.8), *p* = 0.019]. The BED-RIT relationship based on objective measurements resulted in a significant correlation between the alteration in the erythema and hyperpigmentation values and the administered BED, as shown in Fig. 3. The Δerythema and Δpigmentation values increased gradually with increasing BED (*r* = 0.196, *p* = 0.006; *r* = 0.220, *p* = 0.002, respectively). A decreasing trend was also observed in the Δelasticity index with increasing BED; however, the correlation was not significant (*p* = 0.055).

Based on qualitative physician assessment, the RTOG late toxicity grade increased with increasing BED (mean BED: grade 0, 90.8; grade 1, 94.4; grade 2, 105.9, *p* < 0.001).

**Fig. 4:**
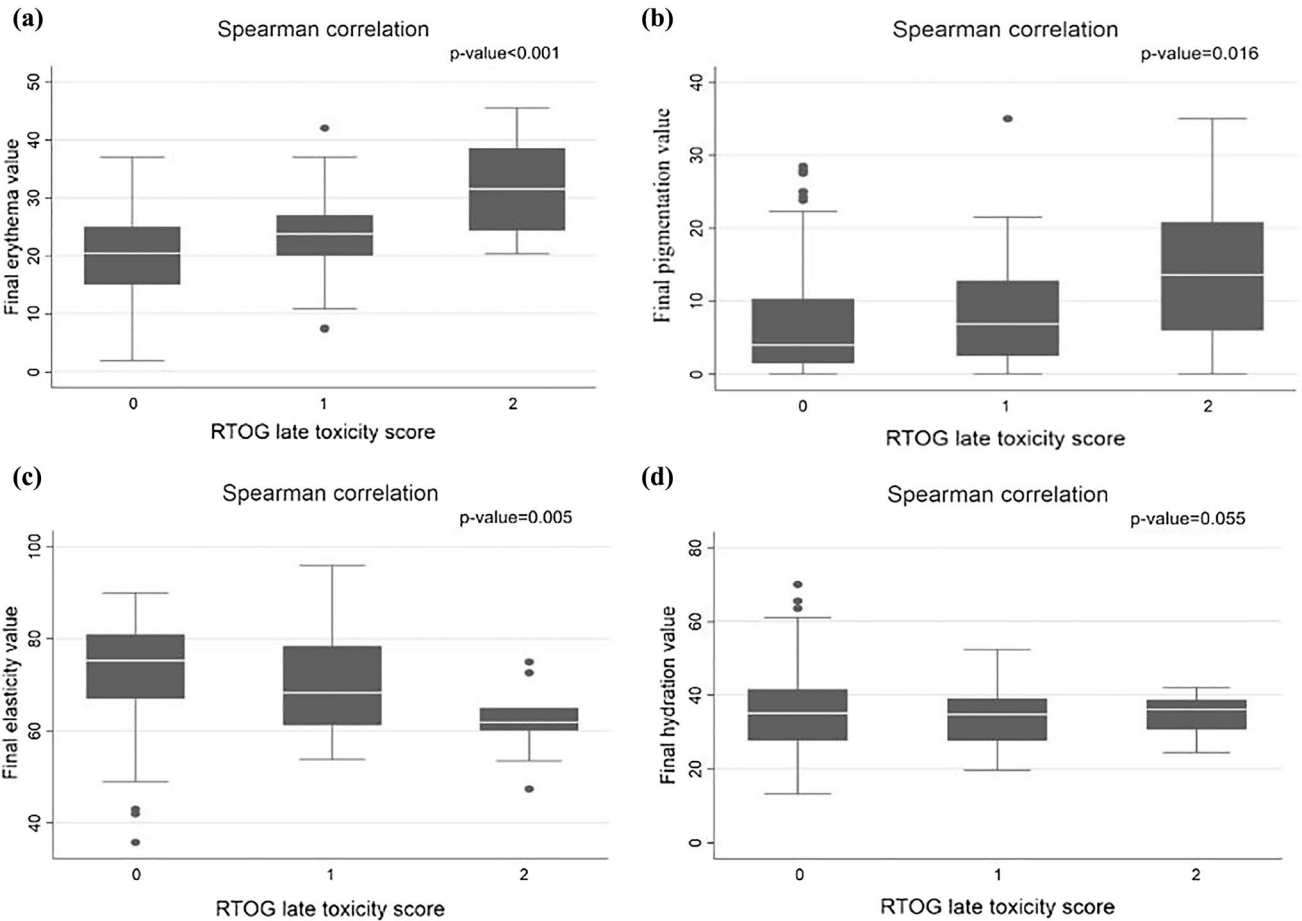
Comparison of erythema (**a**), hyperpigmentation (**b**), elasticity (**c**) and hydration (**d**) with the Radiation Therapy Oncology Group (RTOG) late toxicity score

In comparison of the objective multiprobe evaluation and clinical assessment (RTOG scale), there was an increase in erythema and hyperpigmentation with increasing RTOG skin toxicity grade (*p* < 0.001, *p* = 0.016, respectively) and a decrease in the elasticity index with increasing RTOG subcutaneous toxicity grade (*p* = 0.005) (Fig. 4). Other potential toxicity-related factors, including age, interval time between radiotherapy and toxicity assessment, chemotherapy and hormonal therapy were not correlated with RIT.

## Discussion

Currently, different toxicity scales are used to assess RIT. Despite its speed and simplicity, the measurement of skin reactions usually depends on subjective visual and palpation-based tools. The RTOG and CTCAE scores, although valuable and widely used, have many drawbacks, particularly their lack of objective measures, which carries a considerable risk of intra- and interobserver variability [10]. Especially in multicenter clinical trials, this variability can lead to discrepancies in toxicity outcomes between different institutions and may limit their value as result measures. In addition, it is widely agreed that with the development of various radiotherapy technologies, quantitative assessments are needed to accurately detect the slight changes in RIT caused by these new technologies. Thus, several studies have attempted to measure RIT using quantitative methods (Table 2). Numerous techniques have been developed to objectively assess RIT via the measurement of associated skin characteristics, including ultrasound [11–13], spectrophotometry [14, 20], thermal images [17], LDF [18], mexameter probes [19, 21], viscoelasticity skin analyzers [23], corneometry [19–21] and multiprobe devices, etc. [19, 21, 24–26]. Yoshida et al. [11] tested the reliability of the ultrasonic assessment of radiation toxicity and found that the resulting ultrasound measurements of skin thickness changes were correlated with the RTOG scale score, suggesting that this technique can be used as a reliable method to assess RIT. Unlike our assessment, the use of ultrasound requires long-term training, which is not conducive to its application in clinical practice. Yoshida et al. evaluated radiation dermatitis by a spectrophotometer. CTCAE scales were found to be associated with a* and L* values, which are indicators of skin color alteration [13]. Saednia et al. [17] reported that thermal imaging markers could be used to monitor RIT. Patients with a CTCAE toxicity score > 2 demonstrated a significant increase in skin temperature. In the study by Huang et al. [19] a LDF was used to successfully measure acute radiation dermatitis, and the resulting quantitative values were shown to be correlated with the RTOG, CTCAE and WHO scores. This study also evaluated the pigmentation and skin hydration of the breast through a multiprobe device. Those clinical scoring criteria were ﻿moderate correlated to pigmentation; however, they were not found to be related to moisture analysis. Another study by González et al. [18] also used LDF to monitor acute radiation-induced dermatitis. The results showed that the LDF microcirculation index was correlated with the CTCAE scale score. These technologies have been used mainly in the evaluation of acute toxicity; only a small proportion of objective assessment techniques have been used to monitor late toxicity. Since some acute effects may resolve without chronic sequelae, our research as a measure of late toxicity, may be much better corelated with long-term patient quality of life. In our study, late RIT was assessed by a multiprobe device. We used the color (redness and darkness) of skin as an indicator of erythema and hyperpigmentation, skin elasticity as a surrogate for fibrosis and skin moisture content as an indicator of skin hydration. The treated breasts showed higher erythema and hyperpigmentation and lower elasticity and hydration than untreated breasts. Hydration did not change much after radiation, which maybe due to variability in skin care [3]. Subsequently, we compared clinical assessment measurements with our objective evaluations of RIT, and our results agree with those of the aforementioned studies. Higher erythema, hyperpigmentation and less elasticity are indicators of dermatitis and fibrosis, respectively, the most common signs of late toxicity [3], and were significantly correlated with the RTOG criteria. We suggest that our objective multiprobe measurement system may be used as a reliable clinical tool for assessing RIT. Furthermore, treated breasts with grade 0 RTOG toxicity demonstrated significantly higher values of erythema and hyperpigmentation and lower values of elasticity than the corresponding nonirradiated breasts. These findings indicate the presence of an underlying but invisible or nonpalpable skin change, suggesting that compared with clinical assessment alone, the multiprobe device can demonstrate more reliable changes of erythema, hyperpigmentation and elasticity and hydration. Therefore, our objective measurement tool can be used in the assessment of RIT as a research tool for use in clinical trials and may be more sensitive than the RTOG scale, as it can detect slight changes in RIT that are difficult to determine by visual or tactile examination.

**Table 2:** Studies using quantitative toxicity assessments

Study	*n*	Median follow-up	Radiation schemes	Biophysical parameters	Quantitative techniques	Qualitative assessment
Warszawski et al. [12]	29	*n* = 18: ≤ 3 months; *n* = 11: 30 months	CF: 46–50 Gy/2 Gy	Skin thickness	Ultrasound	RTOG
Yoshida et al. [11]	26	*n* = 8: < 6 months; *n* = 18: ≥ 6 months	CF: 50.0–50.4 Gy/1.8–2.0 Gy	Skin thickness; hypodermal surface; glandular tissue	Ultrasound	RTOG
Landoni et al. [13]	89	20.5 months	HF: 34 Gy/10 Fx/3.4 Gy	Skin thickness	Ultrasound	CTCAE
Wengstrom et al. [16]	53	Acute toxicity (follow-up: N/R)	CF: 50 Gy/2 Gy	Erythema; pigmentation	Spectrophotometer; Measure digital images (Camera)	RTOG
Schmeel et al. [14]	70 in CF; 70 in HF	6 weeks	CF: 50 Gy/25 Fx; HF: 40.05 Gy/15 Fx	Erythema; pigmentation	Spectrophotometer	CTCAE
Yamazaki et al. [15]	46 in CF; 26 in HF	12 months	CF: 50 Gy/25 Fx; HF: 42.56 Gy/16 Fx	Color alteration	Spectrophotometer	CTCAE
Yoshida et al. [20]	118	12 months; subgroup (*n* = 28): 5 years	CF: 48.4–50 Gy/22–25 Fx	Color alteration; skin moisture	Spectrophotometer; Corneometer	CTCAE
Saednia et al. [17]	90	During RT	HF: 42.50 Gy/16 fx	Skin temperature (Dermatitis)	Thermal imaging device	CTCAE
Sanchis et al. [18]	63	3 months	HF: 40 Gy/15 Fx/2.67 Gy	Blood flow (Dermatitis)	LDF	CTCAE
Huang et al. [19]	101	Last day of RT	CF: 50.0–50.4 Gy/1.8–2.0 Gy	Blood flow; pigmentation; hydration; skin pH	LDF; Multi Skin Test Center MC900; Corneometer; Skin pH meter	RTOG; CTCAE; WHO
Sekine et al. [21]	43	1 year	CF: 50 Gy/25 Fx;	Erythema, pigmentation; hydration; skin temperature	Multi-Display Device MDD4; (Corneometer; Tewameter; Mexameter); thermometer	CTCAE
Nuutinen et al. [24]	21	5 weeks; subgroup (*n* = 14): 2 years	CF: 50 Gy/25 Fx;	Dielectric constant (Erythema; fibrosis)	Dielectric constant	
Shumway et al. [25]	80	10 weeks	Total radiation dose: < 40 Gy-66 Gy	Erythema; pigmentation; desquamation	Photographs	Photonumeric scale; CTCAE

*CF* conventional fractionation, *HF* hypofractionation, *RTOG* Radiation Therapy Oncology Group, *CTCAE* common terminology criteria for adverse events, *RT* radiotherapy, *LDF* laser Doppler flowmetry

To compare the RIT following different radiotherapy regimen protocols and design novel radiotherapy schedules in clinical trials, the BED was calculated for different fractionation schemes [6, 7, 22, 27]. In our study, patients were grouped according to the radiation-BED used; since the differences in RIT may be relatively small, this task is not straightforward, and a highly accurate and sensitive assessment tool was required. Therefore, we used the multiprobe device to objectively and quantitatively investigate the underlying relationship between BED and RIT, and the subjective RTOG score was also used to assess toxicity. The results of these objective and subjective methods allowed us to determine the impact of BED on RIT. Regarding the objective assessment, the increase Δerythema and Δpigmentation were correlated with an increase in the delivered BED. Additionally, a decreasing trend was also observed in the Δelasticity index with increasing BED. In terms of the subjective assessment, an increase in the BED resulted in a higher RTOG toxicity grade. Therefore, based on the results of the objective and subjective assessments, we conclude that a lower BED can result in fewer RIT outcomes. This finding has also been confirmed in other clinical trials. Late toxicities were less common in radiation arms with lower BEDs than in those with higher BEDs [4, 28]. This BED-RIT relationship may allow us to make risk–benefit decisions on radiation schedules based on tumor control and possible toxicities. In addition, these results confirm the sensitivity and accuracy of the multiprobe device, which allowed us to detect slight changes in RIT in relation to minor alterations in the radiotherapy schemes used. In future work, this objective assessment may also be used as a monitoring tool to evaluate toxicity resulting from new radiation technologies.

There are several novel radiation schedules that can reduce hospital visits. The use of the FAST-Forward radiation regimen has rapidly increased for selected low-risk patients [5]. This low-BED radiation regimen (BED = 69.1 *α*/*β* = 3.1 Gy) may result in a lower possibility of developing RIT according to our RIT-BED relationship. The COVID-19 pandemic has promoted the adoption of new evidence-based schedules [29], which, in turn, has prompted us to compare different radiation regimens based on the establishment of a more accurate and sensitive evaluation system. Given the accuracy of our objective assessment, the risk and benefits of different treatment schedules can be discussed, facilitating the sharing of decision-making with patients.

Our objective assessment technique offers several advantages. First, the assessment is fast, straightforward, and noninvasive, and the healthcare workers responsible for operating the device only need minimal training. In contrast, some objective assessment techniques, such as ultrasonography, require longer training periods. Second, it can facilitate the detection of slight changes in RIT that are difficult to determine by visual inspections and palpation measurements. Third, this technique can be used in multicenter clinical trials to avoid potential intra- and inter-evaluator biases while facilitating researchers in comparing their results from those of other members of the scientific community. Fourth, given the continuous innovations in several modern radiotherapy techniques (IMRT, etc.) and the increasing number of different radiotherapy regimens (intraoperative radiotherapy, etc.), a continuous and objective scale allows the accurate detection of the development of RIT to improve the effect of new radiotherapy approaches.

A possible limitation of our study may lie in the potential differences in RIT at each time interval. However, in our study, multivariable regression analyses were performed to identify the influence of the interval time factor in both the subjective and objective assessments, and no association was found between RIT and interval time for either radiotherapy or toxicity assessment. In addition, the absence of skin care, as well as individual phenotype, genotype and molecular profiles, may also be considered RIT-related factors [30], but we did not include them in this study. Indeed, among all searched studies, no single factor was shown to be significant, and in certain circumstances, the opposite conclusions were drawn. We expect our sensitive objective assessment tool to provide further clinical evidence and to be used to determine the individual predisposing factors of RIT.

To the best of our knowledge, our series is the largest study to assess late RIT (erythema, hyperpigmentation, elasticity and hydration) using objective and quantitative tool. Moreover, this study also analyzed the BED-RIT relationship using both subjective and objective assessments.

## Conclusions

We found that the Multi Skin Test Center is a noninvasive, useful and sensitive tool for quantitatively monitoring RIT in patients undergoing radiotherapy for breast cancer. The toxicity results measured by this objective assessment are significantly related to the subjective RTOG toxicity score. A higher BED is associated with the development of more severe toxicity.

## Supplementary Information

Below is the link to the electronic supplementary material.

Supplementary file1 Supplementary material figure a: Example of a measure in a patient with a probe (JPG 866 KB)

Supplementary file2 Supplementary material figure b: Multi-probe device Multi Skin Test Center MC750 (JPG 713 KB)

## Data Availability

The datasets generated during and/or analyzed during the current study are available from the corresponding author on reasonable request.
