# Supplementary figures and images for: Quantitative assessments of late radiation-induced skin and soft tissue toxicity and correlation with RTOG scales and biological equivalent dose in breast cancer
Source: Clin Transl Oncol. 2021 Nov 18;24(5):836–45. doi: 10.1007/s12094-021-02729-z (PMC8600910; doi:10.1007/s12094-021-02729-z)

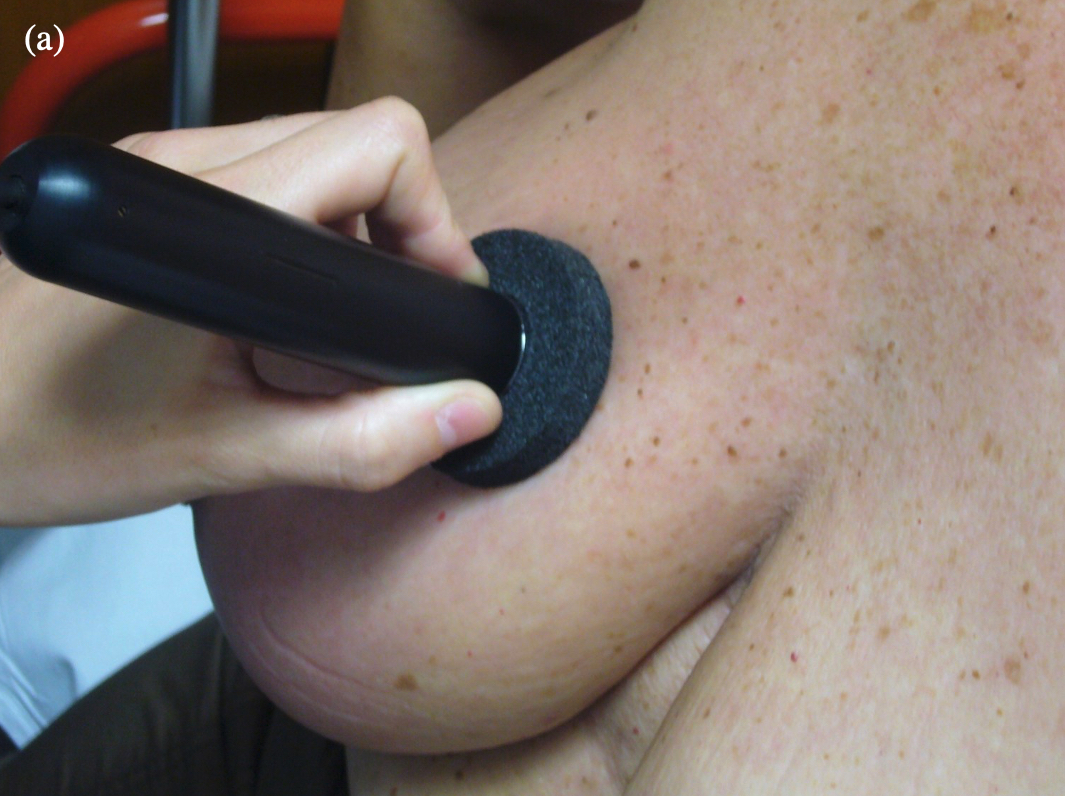

Supplement: Supplementary file 1 — Supplementary file1 Supplementary material figure a: Example of a measure in a patient with a probe (JPG 866 KB) [file 12094_2021_2729_MOESM1_ESM.jpg]

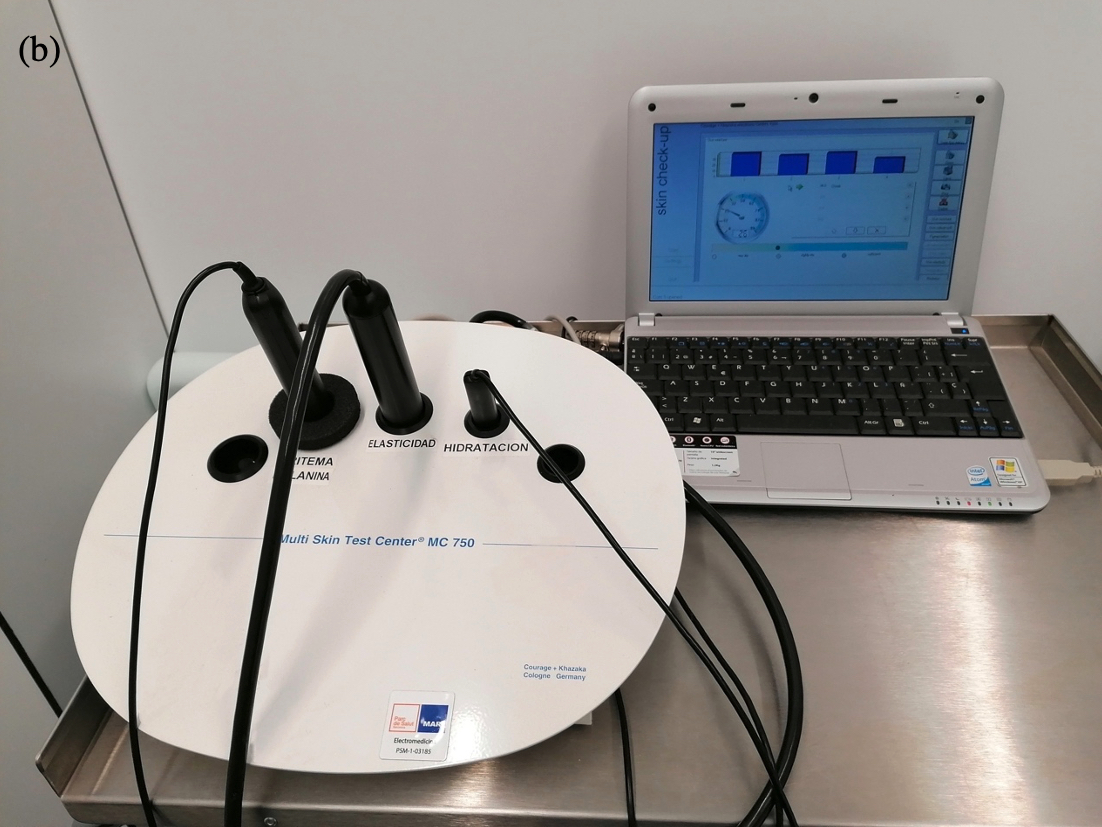

Supplement: Supplementary file 2 — Supplementary file2 Supplementary material figure b: Multi-probe device Multi Skin Test Center MC750 (JPG 713 KB) [file 12094_2021_2729_MOESM2_ESM.jpg]
